# Global Transcriptional Differences in *Staphylococcus aureus* Biofilm-Associated Genes in a *brpR* Mutant Compared to Wild-Type Strain

**DOI:** 10.3390/antibiotics15080787

**Published:** 2026-08-14

**Authors:** Hailey Dyce, Paul Schweiger, Robin Patel, Stephen Johnson, Isabelle Sharp, William R. Schwan

**Affiliations:** 1 Department of Microbiology, University of Wisconsin-La Crosse, La Crosse, WI 54601, USA; hdyce@mcw.edu (H.D.); pschweiger@uwlax.edu (P.S.); isabellesharp22@gmail.com (I.S.); 2 Division of Clinical Microbiology, Department of Laboratory Medicine and Pathology, Mayo Clinic, Rochester, MN 55905, USA; patel.robin@mayo.edu; 3 Department of Quantitative Health Science Research, Mayo Clinic, Rochester, MN 55905, USA

**Keywords:** BrpR, BrpS, SK-03-92, *Staphylococcus aureus*, biofilms, late-stage competence, peptidoglycan

## Abstract

**Background**: *Staphylococcus aureus* causes bloodstream and skin infections in humans. The prevalence of multidrug-resistant *S. aureus* strains means new antibiotics are needed. A novel antimicrobial drug named SK-03-92, a synthetic aromatic organic stilbenoid compound, kills *S. aureus* cells within 30 min, but an increase in both biofilm formation and persister cells occurs. SK-03-92 treatment downregulates transcription of the biofilm regulating protein regulator (*brpR*) gene and biofilm regulating protein sensor (*brpS*) gene in *S. aureus*. BrpR/BrpS system may be a LytTR regulatory system tied to biofilm formation, creation of persister cells, and late-stage competence in *S. aureus*. The aim of this study was to determine what biofilm, late-stage competence, and persister-associated genes were regulated in a *brpR* mutant compared to wild-type strains. **Methods**: In this study, involvement of BrpR in regulating other genes was assessed by comparing transcriptional changes in a *brpR* mutant strain to the *S. aureus* parent strain via RNA sequencing (RNA-Seq). Bioinformatic analysis was then performed on the RNA-Seq data to assess what biochemical pathways might be involved. **Results**: From these analyses, 440 genes were identified that had significant differences in transcript abundance when comparing the *brpR* mutant to wild-type strains. Quantitative reverse transcription polymerase chain reaction analysis confirmed *bacA*, *icd*, *metE*, and *pdhA* transcript levels were lower, whereas *alr* and *mraY* were higher in the *brpR* mutant versus wild-type strain. Furthermore, an enzymatic assay targeting NADH production from the pyruvate dehydrogenase complex showed lower levels in the mutant compared to wild-type strain. **Conclusions**: Overall, the study demonstrated several biosynthetic pathways tied to biofilm formation and late-stage competency may be regulated by BrpR and some potential leads for the mechanism of action of the SK-03-92 drug were uncovered.

## 1. Introduction

*Staphylococcus aureus* is an opportunistic bacterial pathogen found in the nasopharynx of about 20% of healthy humans [1]. Besides being part of the human normal microbiota, *S. aureus* is the leading cause of skin and soft tissue infections as well as endocarditis in humans [2,3,4]. A combination of increasing antibiotic resistance within the bacteria and formation of biofilms in humans has made treatment more challenging [5,6]. Thus, new antibiotics are needed to treat *S. aureus* infections.

To address the antibiotic resistance issue, a (E)-3-hydroxy-(5)-methoxystilbene compound found in sweet fern plants (*Comptonia peregrina*) was isolated and tested for antimicrobial activity [7]. From this compound, several analogs were synthesized, including a (E)-3(2-(benzo[b]thiophen-2-yl)vinyl)-5-methoxyphenol analog named SK-03-92. The SK-03-92 small molecule exhibited antimicrobial activity against all tested Gram-positive bacteria, including several strains of *S. aureus*, with MICs of 1–2 µg/mL [8].

Although SK-03-92 can kill *S. aureus* cells within 30 min [8], its mechanism of action is currently unknown. An initial RNA microarray comparing transcriptional regulation in *S. aureus* treated with SK-03-92 versus untreated cells was performed to try to elucidate the mechanism of action for SK-03-92. The two most downregulated genes in SK-03-92 treated *S. aureus* cells were two uncharacterized genes, *MW2284* and *MW2285* [9]. From the initial annotation, *MW2284* was proposed to encode a transcriptional regulator protein, while *MW2285* was proposed to encode a membrane sensor kinase. Through initial bioinformatic analysis, both proteins were originally hypothesized to be members of a two-component system (TCS) [9]. However, later bioinformatic analyses showed that both proteins likely comprise a LytTR regulatory system (LRS) [10,11]. Among Gram-positive bacterial species, LytTR superfamily proteins are conserved and serve to regulate virulence factor gene transcription that includes autolysis [12].

Besides quickly killing *S. aureus* cells, SK-03-92 also caused greater biofilm formation in treated compared to untreated *S. aureus* cells [13]. SK-03-92 drug is a plant-derived phytoalexin, a class of small molecules class known to decrease sortase A activity [14,15,16]. Sortase A is important in both adherence to a solid surface during the first step of biofilm formation and dispersion [17,18,19]. To further understand the function of the MW2284 and MW2285 proteins in biofilm formation, *MW2284* and *MW2285* mutants were assessed for *srtA* transcription level changes using quantitative reverse transcriptase–polymerase chain reaction (qRT-PCR). The *MW2284* and *MW2285* mutants had 9.2-fold and 8.1-fold higher *srtA* transcript abundance than wild-type strains, respectively, suggesting the MW2284/MW2285 LRS may be repressing *srtA* transcription [13]. Furthermore, higher biofilm formation was observed in the *MW2284* and *MW2285* mutant strains compared to wild-type strain Newman, suggesting the MW2284/MW2285 LRS repressed biofilm formation in *S. aureus*. Because of the association of MW2284 and MW2285 with biofilm formation, the *MW2284* gene was named *brpR* (biofilm regulating protein regulator) and *MW2285* was named *brpS* (biofilm regulating protein sensor) [13].

Another effect of SK-03-92 treatment was an elevation in antibiotic tolerance and the number of SK-03-92 associated persister cells [9]. Persister cells are metabolically inactive cells that stop growing in the presence of a particular antibiotic, but regain their ability to grow and be antibiotic susceptible once exposure to the drug is halted [20]. After an initial rapid kill phase, a small percentage of the dormant population remains alive in the form of persister cells [21]. Moreover, formation of biofilms is directly linked to persister cell formation and induction of late-stage competency, a stress response allowing the cell to uptake DNA from its environment [22]. When a biofilm is formed, there is accumulation of a competence stimulating peptide due to high population density, leading to induction of late-stage competency [23]. Decreased metabolism during late-stage competency while bacterial cells are in a biofilm can lead to the formation of persister cells [22]. Therefore, the increase in persister cell populations in SK-03-92 treated *S. aureus* cells *versus* untreated cells suggests SK-03-92 treatment may lead to an increase in late-stage competency.

We propose the BrpR/BrpS system is a LRS that transcriptionally regulates several genes that encode proteins tied to late-stage competency and biofilm formation in *S. aureus*. The aim of the study was to identify genes with transcript abundance differences in the *brpR* mutant versus wild-type strains, particularly those genes that may be tied to encoding gene products important for biofilm formation, late-stage competence or creation of drug persister cells. This study shows several biosynthetic pathway genes are affected at the transcriptional level by a *brpR* mutation; they may, in turn, be tied to the SK-03-92 mechanism of action.

## 2. Results

### 2.1. RNA Sequencing (RNA-Seq) and Bioinformatic Analyses Demonstrate Several Pathways Affected by a brpR Mutation

To elucidate the potential regulatory function of the BrpR/S LRS in *S. aureus* and attempt to narrow potential targets of the SK-03-92 drug, RNA sequencing (RNA-Seq) was performed comparing *S. aureus* strain Newman wild-type to a *brpR* mutant strain. A total of 440 genes had significant transcript abundance differences (≥1.0 log_2_-fold change) out of 2697 total genes in wild-type *S. aureus* strain Newman *versus* the *brpR* mutant, representing 16% of the transcriptome [24]. For the 440 genes, transcript abundance increased for 182 genes (41%) and decreased for 258 genes across the whole strain Newman genome (59%; Supplementary Table S1). Moreover, these genes mapped to several different pathways specific for KEGG metabolic pathways (Table 1).

Genes identified as having significant transcript abundance differences through the bioinformatic analysis of the RNA-Seq results revealed many KEGG metabolic pathways affected by the *brpR* mutation. Some of the pathways that had the highest number of transcriptionally dysregulated genes included the TCA cycle; the phosphotransferase system; quorum sensing; TCSs; ABC transporters; carbon metabolism; pyruvate metabolism; peptidoglycan biosynthesis; D-amino acid metabolism; phenylalanine, tyrosine, and tryptophan biosynthesis; cysteine and methionine metabolism; and purine metabolism (Table 1).

### 2.2. Quantitative Reverse-Transcribed Polymerase Chain Reaction (qRT-PCR) Analysis-Confirmed Transcript Abundance Differences Associated with Genes Tied to Biofilm Formation

To confirm some of the RNA-Seq results, qRT-PCR was performed on six genes that were upregulated or downregulated as a result of the *brpR* mutation when compared to wild-type *S. aureus*. The six genes chosen encode products involved in the following pathways: the TCA cycle/pyruvate dehydrogenase complex (*icd* and *pdhA*), peptidoglycan synthesis (*alr*, *bacA*, and *mraY*), and methionine synthesis (*metE*). Genes associated with the pyruvate dehydrogenase complex, TCA cycle, and peptidoglycan synthesis were chosen because of their gene products’ hypothesized effects on biofilm formation.

**Figure 1:**
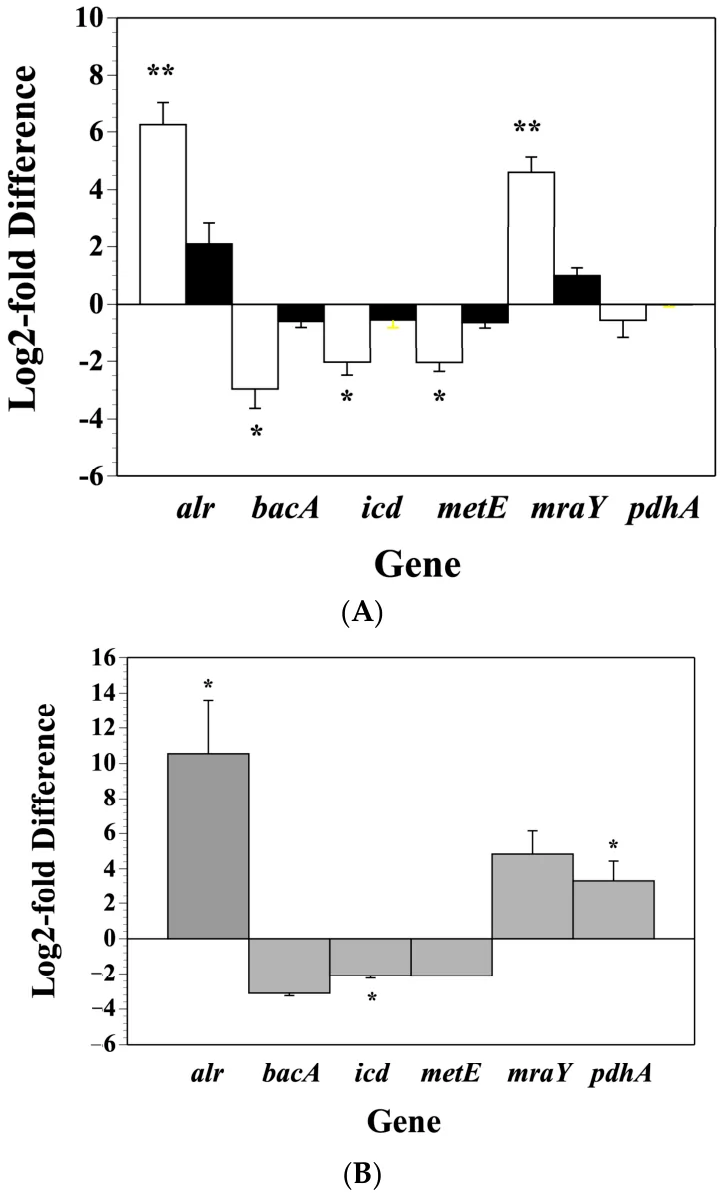
Quantitative reverse-transcribed polymerase chain reaction results of (**A**) *S. aureus* strain Newman *alr*, *bacA*, *icd*, *metE*, *mraY*, and *pdhA* transcription in wild-type bacteria (standardized to 0) compared to a *S. aureus* strain Newman *brpR* mutant (white column) and the complemented *brpR* mutant (black column) and (**B**) *S. aureus* strain JE2 (standardized to 0) compared to strain NE671 (*brpR* mutant gray column). The data represents the mean ± SD from three separate runs. * indicates *p* ≤ 0.05 statistically significant transcript abundance differences and ** indicates *p* ≤ 0.001 statistically significant transcript abundance differences between wild-type and the *brpR* mutant.

All of genes targeted through qRT-PCR displayed results similar to those observed using RNA-Seq (Figure 1A) using a strain Newman background. In the *brpR* mutant strain *versus* wild-type, *alr* and *mraY* were upregulated 6.25-fold and 4.60-fold, respectively; whereas *bacA*, *icd*, *metE*, and *pdhA* were downregulated −2.76-fold, −2.03-fold, −2.04-fold, and −0.55-fold, respectively. Complementation with the pAB1-5 plasmid [25] restored transcript abundance levels to near-wild-type levels.

To demonstrate that effects of the *brpR* mutation was not confined to the Newman strain background, qRT-PCR was also done comparing the well characterized JE2 strain to its *brpR* mutant strain NE671. In the *brpR* mutant *versus* wild-type strain, *alr* and *mraY* were upregulated 10.54-fold and 4.84-fold, respectively, demonstrating results similar to what was observed for the Newman strain background (Figure 1B). For genes that displayed less transcript abundance in the mutant *versus* wild-type, the *bacA*, *icd*, and *metE* showed −3.10-fold, −2.10-fold, and −2.11-fold changes that also mimicked the trends shown for the Newman strain. However, transcript abundance for the *pdhA* gene was 3.13-fold higher in JE2, a marked difference from the Newman strain.

### 2.3. Pyruvate Dehydrogenase Complex Activity Is Lower in the brpR Mutant Versus Wild-Type Strain

**Figure 2:**
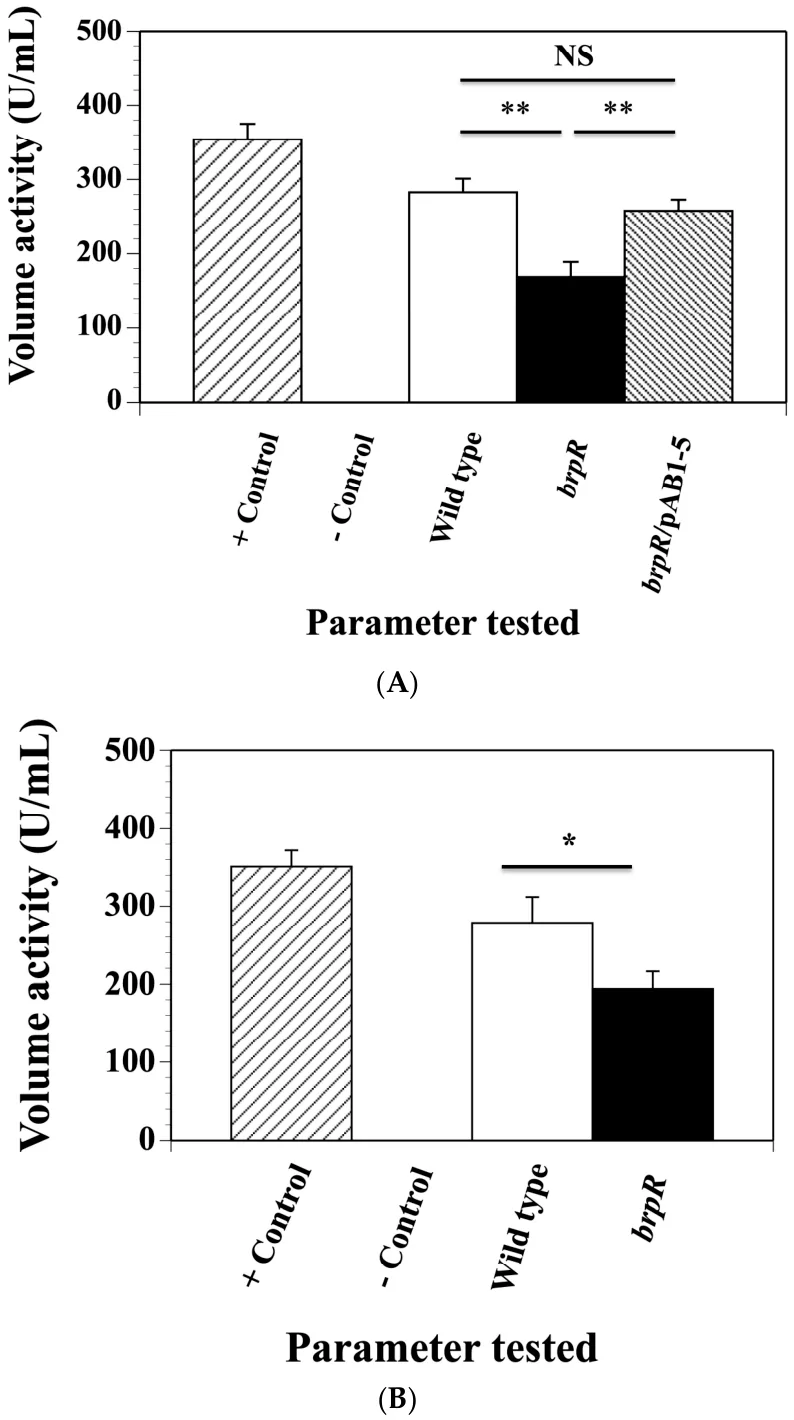
Pyruvate dehydrogenase complex activity in (**A**) wild-type *S. aureus* strain Newman compared to a *brpR* mutant strain and complemented *brpR* mutant (*brpR*/pAB1-5) and (**B**) wild-type *S. aureus* strain JE2 compared to a *brpR* mutant strain. *Escherichia coli* served as a positive control (+Control) and no cells added acted as a negative control (−Control). The values represent the mean ± standard deviation from three separate trials with * *p* < 0.05, ** *p* < 0.01, and NS = not significant.

Because the qRT-PCR results did not fully support the RNA-Seq results, a pyruvate dehydrogenase assay was performed to clarify regulation of the pyruvate metabolism related *pdhA* gene. *Escherichia coli* was used as a positive control because of its known pyruvate dehydrogenase complex activity [26], whereas a sample with no bacterial cells added served as the negative control. In the strain Newman background, there was significantly less pyruvate dehydrogenase activity for the *brpR* mutant (169 ± 20 U/mL) compared to the wild-type (283 ± 18 U/mL). Moreover, complementation of the *brpR* mutant with the pAB1-5 plasmid brought the activity to a near-wild-type level (258 U/mL). Significantly lower average pyruvate dehydrogenase activity was seen for the *brpR* mutant (193.96 ± 22.22 U/mL) compared to the wild-type (278.71 ± 33.44 U/mL; Figure 2). These data correlate with the expression changes seen in the RNA-Seq (Supplementary Table S1) and qRT-PCR results for the Newman strain (Figure 1A) but not the qRT-PCR results for strain JE2 (Figure 1B).

## 3. Discussion

Antibiotic-resistant *S. aureus* has become a huge problem worldwide as treatment options continue to decline [5,27]. To combat drug-resistant bacteria, the antimicrobial drug SK-03-92 was developed. However, its mechanism of action is still unknown. An RNA microarray performed on SK-03-92-treated *S. aureus versus* untreated *S. aureus* cells showed that the two most downregulated genes were *MW2284* and *MW2285*, now named *brpR* and *brpS*, respectively [9,13]. It was hypothesized that the BrpR protein forms an LRS with BrpS to transcriptionally regulate genes that encode products that may decrease late-stage competency and biofilm formation. This current study looked further into what the BrpR protein encoded by the *brpR* gene may globally regulate in *S. aureus*.

To determine more broadly which genes might be regulated by the BrpR/S putative LRS, RNA-Seq was performed comparing a *S. aureus brpR* mutant to wild-type *S. aureus* of the transcriptional changes observed through the RNA-Seq and confirmed using qRT-PCR analyses, those tied to peptidoglycan synthesis may have the most relevance in biofilm formation as well as be a potential mechanism of action of SK-03-92 killing. The *mraY* gene encodes the MraY protein, which is a phospho-N-acetylmuramoyl-pentapeptide-transferase. This enzyme links the pentapeptide chain attached to activated NAM to undecaprenyl-phosphate to form Lipid I, which is used to transport the NAM pentapeptide outside of the cell [28]. More MraY protein would likely lead to an increase in transport of the peptidoglycan precursor outside of the cell. On the other hand, *bacA* encodes an undecaprenyl-diphosphate phosphatase that removes a phosphate from undecaprenyl-diphosphate to make undecaprenyl-phosphate [29]. Downregulation of this gene indicates there may be a decrease in flipping peptidoglycan precursors outside of the cell. The transcriptional regulation of *mraY* and *bacA* suggest an imbalance of the bactoprenol cycle with MraY overexpression increasing the demand for undecaprenyl-phosphate and BacA repression decreasing the supply of recycled undecaprenyl-phosphate. Consequently, undecaprenyl-phosphate will become limiting with increased undecaprenyl-diphosphate and lipid-lined intermediates. Peptidoglycan synthesis would become inefficient despite increased initiation, stalling at the Lipid I or Lipid II step. This would lead to cell envelope stress that could lead to cell lysis and the release of more extracellular DNA.

Alr is an alanine racemase that converts L-alanine into D-alanine [30]. D-Alanine is one of the amino acids in the pentapeptide chain that forms the cross-linkages between NAM molecules in *S. aureus*. *S. aureus* cells and those cells in a biofilm typically have a thicker cell wall, so more D-alanine is needed to create a thicker peptidoglycan layer [31]. D-Alanine is also commonly added to teichoic acids via ester linkages (i.e., D-alanylation) in *S. aureus* cell walls, which introduces cationic amines (NH_3_^+^). This positive charge is necessary for *S. aureus* cells to attach to surfaces by forming cationic bridges between the negatively charged cell surface and an attachment surface, which is also typically negatively charged, in the first step of biofilm formation. Consequently, a decrease in D-alanine can cause reductions in biofilm formation [31,32]. Both the RNA-Seq and qRT-PCR analyses showed higher transcript abundance that would suggest more Alr protein is made to contribute to the thicker peptidoglycan layer observed in biofilms.

Two recent publications have shown that a *brpS* mutation (*SAUSA300_2311*) in the strain JE2 background was linked to alterations in the peptidoglycan structure in *S. aureus* [33,34]. The one paper examined resistance to the MEL-B natural product and found the *yjbI*, *clpP*, *pbuX*, and *brpS* mutants were resistant to the MEL-B small molecule [33]. From the RNA-Seq analysis in this study, transcript abundance differences were seen for *yjbI* and *clpP* when comparing the *brpR* mutant to wild-type, suggesting BrpR might regulate transcription of both genes. Furthermore, Scafidi et al. [34] demonstrated that a *brpS* mutation spatially changed the *S. aureus* peptidoglycan layer. Since we believe that BrpS is part of an LRS with BrpR, these studies support the idea that regulation of peptidoglycan layer-associated genes and their gene products could lead to lysis of the bacteria as a mechanism of action of SK-03-92 killing, as well as allow greater biofilm formation in those cells that survive the initial exposure to SK-03-92.

Another explanation for the increase in biofilm formation in the *brpR* mutant compared to the wild-type is the lower transcript abundance of all three genes (*pdhA*, *pdhC*, and *lpdA*) that encode the enzymes that make up the pyruvate dehydrogenase complex. Downregulation of these genes would likely result in a decrease in the conversion of pyruvate to acetyl-CoA that can be used for the TCA cycle and therefore a decrease in energy production, which is characteristic of biofilms and formation of persister cells [6,32,35].

The pyruvate dehydrogenase assay supported the RNA-Seq and qRT-PCR results in the strain Newman background, but not the qRT-PCR results from strain JE2. Strain differences could explain this discrepancy. Moreover, there could be post-transcriptional regulation occurring that affected the activity. In addition, the pyruvate dehydrogenase assay supported the qRT-PCR results from a previous study that showed a two-fold decline in *lrgA* transcription in a *brpS* mutant compared to wild-type [13]. LrgA acts not only as an antiholin but also as a pyruvate transporter under microaerophilic and anaerobic conditions [36,37,38]. BrpR/S LRS transcriptional regulation of the *lrgA* gene could disrupt the antiholin–holin equilibrium, leading to cell death, as well as affect pyruvate transport in the *S. aureus* cells, leading to a transition to a dormant state.

Besides the indirect effect caused by a diminishment of the pyruvate dehydrogenase complex, another energy drain would be a disruption of the TCA cycle. Three genes (*icd*, *sucB*, and *fumC*) that encode enzymes in the TCA cycle had lower transcript abundance in the *brpR* mutant compared to wild-type strains. CitC is an NADP-dependent isocitrate dehydrogenase encoded by the *icd* gene that converts isocitrate into α-ketoglutarate [39], and in turn, make amino acids like glutamine and glutamate essential for cell survival, as well as biofilm formation and maintenance [40]. Glutamine levels change with nitrogen availability, signaling cells to begin biofilm formation when low nitrogen conditions occur [41,42]. Lower levels of α-ketoglutarate should in turn lead to a decrease in glutamine production, lower nitrogen availability, and increased biofilm formation in the *S. aureus brpR* mutant compared to wild-type strains.

Subsequently, SucB is a dihydrolipoyllysine-residue succinyltransferase that converts α-ketoglutarate into succinyl-CoA and FumC is a Class II fumarate hydratase that converts fumarate into malate. Both of these steps are accompanied by the reduction in an electron acceptor (i.e., NAD^+^ or FAD), which can later donate electrons to the electron transport chain and be used to make ATP [39]. Taken together, the reduced transcription of *icd*, *sucB*, and *fumC* are predicted to reduce the overall TCA cycle activity, which shifts metabolism toward a biofilm-promoting, dormant persister state [43].

Besides regulation of genes that are tied to the TCA cycle, transcriptional changes in genes involved in methionine synthesis could also impact biofilm formation. All four genes (*metE*, *MW_RS01765*, *MW_RS01760*, and *MW_RS01755*) that encode enzymes used to create methionine from L-cysteine or L-homoserine had lower transcript abundance in the *brpR* mutant as shown using RNA-Seq analysis. Synthesis of methionine is a key step for the synthesis of S-adenosylmethionine (SAM), the cell’s primary methyl donor [44]. Decreased methionine synthesis will lead to decreased SAM production, which is important for autoinducer-2 production used for quorum sensing associated with the regulation of biofilm formation.

A decrease in methionine production would not only affect SAM production, but general translation as well. Methionine is the start codon used to initiate translation and a decrease in methionine levels would lead to a decrease in overall translation [45]. This may be happening because of the proposed decrease in energy production described above. When the cell is in a biofilm or a persister state, there is a decrease in metabolism and a decreased need for enzymes used in metabolism to be produced [6,20]. A recent study showed that the SK-03-92 treatment led to a change in MetE levels [11].

This study broadly shows that BrpR may regulate genes tied to biofilm formation, competence, and persister cell formation. Changes in gene regulation related to peptidoglycan synthesis, energy production, and amino acid synthesis indicate increases in biofilm formation, persister cells, and competence because of a *brpR* mutation. The downregulation of genes that encode products involved in energy production would likely create the characteristic decreased energy production commonly seen in persister cells and biofilms. These results support previous research that has shown an increase in biofilm formation and persister cells in a *brpR* mutant versus wild-type and suggest a potential mechanism of action for SK-03-92 may be a disruption of the peptidoglycan layer.

## 4. Materials and Methods

### 4.1. Bacterial Strains, Plasmids, and Media

For RNA-Seq, *S. aureus* strain Newman [46] and a *brpR* mutant of this strain created by transduction of the ϕ80α bacteriophage from *S. aureus* strain JE2 mutant NE671 into *S. aureus* strain Newman were used [13]. For qRT-PCR, strains Newman and JE2 [47] served as the wild-type strains, whereas wild-type *S. aureus* strain JE2 was also used for the pyruvate dehydrogenase assay in this study. The Newman *brpR* and NE671 *brpR* mutant of strain JE2 [47] have a *mariner* transposon insertion into the *brpR* gene and were used in both qRT-PCR and the pyruvate dehydrogenase assays. Strain Newman *brpR*/pAB1-5 contains the pAB1-5 plasmid [25] with a pTGComTcGm2 backbone [48] carrying the *brpR* gene and was selected on BHI media containing 5 mg/mL of tetracycline (Sigma-Aldrich, St. Louis, MO, USA). All media was purchased from ThermoFisher Scientific (ThermoFisher Scientific, Pittsburgh, PA, USA). Wild-type strains were grown on brain heart infusion (BHI) agar statically at 37 °C or in BHI broth (BD BBL, Sparks, MD, USA) shaken at 250 rpm at 37 °C. The *brpR* mutant strains were grown in BHI media with 5 μg/mL of erythromycin (Sigma-Aldrich, St. Louis, MO, USA) with the same incubation conditions. Strain Newman *brpR*/pAB1-5 was grown in BHI media and 5 μg/mL of tetracycline (Sigma-Aldrich).

### 4.2. Isolation of Total RNA

RNA was isolated from each culture using a High Pure RNA isolation kit (Roche Diagnostics, Indianapolis, IN, USA) following the manufacturer’s instructions with one change. The wild-type and mutant strains were grown in 5 mL BHI broth at 37 °C and shaken at 250 rpm overnight, with a second incubation of 100 μL of growth in 10 mL of broth for 2 h when an optical density (OD) between 0.15 and 0.20 was reached. At the first step, 75 μL of 10 mg/mL lysostaphin (ThermoFisher Scientific, Pittsburgh, PA, USA) was added to help disrupt the staphylococcal bacterial cell walls [13]. Each RNA preparation was treated with DNase I (Roche Diagnostics) to remove contaminating DNA. The RNA concentration of each sample was assessed using a NanoDrop 2000 spectrophotometer (ThermoFisher Scientific) at a wavelength of 260 nm and the relative purity was assessed using the 260 nm/280 nm ratio. Each RNA was considered a useable sample if the 260/280 ratio was greater than 1.8 and was then stored at −80 °C.

All RNAs were checked for DNA contamination by polymerase chain reaction (PCR) on each sample using the SaFtsZ1/2 primer pair [49] and an Applied Biosystems 2720 thermal cycler (Applied Biosystems, Waltham, MA, USA). Each PCR had Wigler’s buffer [17 mM Tris-HCl, 16 mM NH_4_SO_4_, 2 mM MgCl_2_, 0.07% β-mercaptoethanol (BioRad Laboratories, Hercules, CA, USA), and 0.1 mg/mL bovine serum albumin (Sigma-Aldrich)] [50], 200 μM dNTPs (Promega Corporation, Madison, WI, USA), 50 pmol each of the SaFtsZ1 and SaFtsZ2 primers (Table 2; Integrated DNA Technologies, Coralville, IA, USA), five units Taq polymerase (Promega Corporation, Madison, WI, USA), 1 μL of RNA with a concentration greater than 200 ng/μL, and water added to make a 50 μL total volume. The PCR amplification conditions were 30 cycles of 95 °C for 1 min, 57 °C for 1 min, and 72 °C for 1 min. Positive and negative controls were JE2 chromosomal DNA and a no-template, respectively. Aliquots of each PCR were separated using a 1.5% agarose gel (ThermoFisher Scientific) containing ethidium bromide (ThermoFisher Scientific). Absence of a *ftsZ* PCR product of 484 base pairs indicated no DNA contamination.

If a PCR product was observed for any sample, the RNA was treated with 20 μmols of Turbo DNase (Invitrogen, Waltham, MA, USA) for 30 min at 37 °C. The RNA was then extracted with an equal volume of phenol (ThermoFisher Scientific) and centrifuged at 12,303× *g* for 10 min at 4 °C using a Sorvall Legend Micro 21R centrifuge (ThermoFisher Scientific). An equal volume of chloroform (ThermoFisher Scientific) was added to the supernatant and centrifuged under the same conditions described above. To precipitate the RNA, two volumes of 100% ethanol (ThermoFisher Scientific) and 1/10 volume of sodium acetate in diethylpyrocarbonate (DEPC)-treated water (Sigma-Aldrich) was added to the sample and incubated at −20 °C overnight. After pelleting the samples using centrifugation at 12,300× *g* for 10 min at 4 °C, 400 µL of 70% ethanol in DEPC-treated water was added to the pellet then centrifuged again as described above. The pellet was then dried and resuspended in 20 µL of DEPC-treated water and the purity of RNA in the sample checked again as described above.

### 4.3. RNA Sequencing (RNA-Seq)

To determine the function of the *brpR* gene in *S. aureus*, an RNA-Seq analysis was performed at the Mayo Clinic core laboratory using RNA isolated from wild-type *S. aureus* strain Newman and a *brpR* mutant in this background. RNA was isolated using the method described above. The RNA-Seq analysis was done on a HiSeq 2500 Illumina platform (Illumina, San Diego, CA, USA) with three RNA samples from each strain. Preliminary analysis of upregulated and downregulated genes was performed by the Mayo Clinic core laboratory.

Transcript quantification was performed using kallisto (v0.46.2) with *S. aureus* strain MW2 used as a reference (accession number GCF_000011265.1; 20). Gene annotations were extracted from the NCBI GFF3 file, and transcripts were mapped to genes using annotated gene names or locus tags. Differential abundance was performed in R (v4.6.1) using the sleuth (v0.30.1) and DESeq2 (v1.34.0) packages. For sleuth, transcript abundances were aggregated to the gene level. A likelihood ratio test compared expression across both conditions (Newman and *brpR* mutant), and Wald tests performed pairwise comparisons. Genes with q-value ≤ 0.05 were considered significant. For differential expression analysis with DESeq2, kallisto transcript abundances were imported and summarized to gene-level count estimates using tximport, followed by pairwise comparison (Newman versus *brpR* mutant). Genes with adjusted *p*-value < 0.05 and an absolute [log_2_-fold change] > 1 were considered significantly differentially expressed.

PCA plots assessed sample clustering across all samples. Volcano plots from DESeq2 visualized differential expression for each pairwise comparison. Bootstrapped TPM distributions from sleuth were used to visualize expression and technical variance for individual genes of interest across conditions.

### 4.4. Bioinformatic Analyses

Genes identified by the Mayo Clinic as statistically significant from the RNA-Seq results were matched to their KEGG ID and listed in a .txt file. The pathview package in R was used to download the pathways for *S. aureus* strain MW2 found in KEGG as xml files that could be uploaded to Cytoscape version 3.10.1 [51,52,53,54,55,56,57]. In Cytoscape, KEGGscape and cytoKEGG plugins were used to import the xml files generated from the pathview package in R into Cytoscape (26, https://github.com/jmvillaveces/cytokegg, URL accessed 2 February 2025). The log_2_-fold change between the mutant versus wild-type and *p*-value was added to Cytoscape by matching the KEGG nodes from the data file containing the log_2_-fold changes and *p*-values to the KEGG nodes in Cytoscape.

### 4.5. cDNA Synthesis

Each RNA preparation was converted to complementary DNA (cDNA) using a First Strand Synthesis kit (Invitrogen, Waltham, MA, USA) following manufacturer’s instructions. Random hexamer primers were used to create the cDNAs from 2 μg of total RNA. The cDNA samples were stored at −80 °C until they were used.

### 4.6. Quantitative Reverse Transcriptase–Polymerase Chain Reaction (qRT-PCR) Analysis

From the bioinformatic analyses noted above, several genes were identified that may have roles in late-stage competence or biofilm formation in *S. aureus*. Six of these genes—*alr*, *bacA*, *icd*, *metE*, *mraY*, and *pdhA* (Table 2)—were targeted using the qRT-PCR analysis to confirm whether the *brpR* mutation resulted in either a significant decrease or increase in transcript abundance versus the parent strain when compared to preliminary RNA-Seq.

The qRT-PCR analysis was done using an iTaq Universal SYBR green kit (BioRad Laboratories, Hercules, CA, USA) according to the manufacturer’s instructions. A 1.5 μL volume of cDNA from either the Newman strain paired with the Newman *brpR* mutant and complemented Newman *brpR*/pAB1-5 strain or the JE2 paired with the NE671 *brpR* mutant strain was added to separate tubes in duplicate. In addition, a no-template (negative control), an RNA sample (negative control), and chromosomal DNA (positive control) were amplified in duplicate. One set of tubes had 50 pmol each of oligonucleotide primer (SaFtsZ1 and SaFtsZ2) targeting the *ftsZ* housekeeping gene to standardize transcript abundance levels for each tube and the same reagents noted above. The other sets of tubes contained 50 pmol each of the two oligonucleotide primers targeting one of the six other genes (Table 2). The PCR conditions included an initial denaturation step at 95 °C for 5 min, then cycling at 95 °C for 30 s, 57 °C for 30 s, and 72 °C for 30 s for a total of 35 cycles followed by a final elongation step at 72 °C for 5 min on a CFX Connect Real-Time system thermocycler (Bio Rad Laboratories). The qRT-PCR data was analyzed using the 2^−ΔΔCT^ method by standardizing the gene of interest transcript levels to *ftsZ* transcript levels [58]. A total of three biological replicates were done for each strain and each of the six genes. Means and standard deviations were calculated for the three replicates for each gene using Excel and a Student’s *t*-test was performed to measure statistical significance with a *p*-value < 0.05 considered statistically significant.

### 4.7. Pyruvate Dehydrogenase Assay

A modified pyruvate dehydrogenase assay was performed to provide a functional assay to support the qRT-PCR results for the *pdhA* gene [26]. Single colonies of the wild-type and the *brpR* mutant strains grown on BHI agar were transferred to 5 mL of BHI broth and incubated with shaking at 250 rpm overnight at 37 °C in triplicate. The samples were subcultured using 100 μL aliquots into 10 mL BHI broth and incubating for 2 h while shaken at 250 rpm at 37 °C. The samples were pelleted using centrifugation for 10 min at 8000× *g* at 4 °C. The pellets were resuspended in 200 μL 0.05 M potassium phosphate buffer, pH 6.6 (0.04 M KH_2_PO_4_, 10 mM K_2_HPO_4_, and 40 mL ddH_2_O with pH adjusted to 6.6 with NaOH or HCl and volume brought to 50 mL).

To lyse *S. aureus*, 75 μL of 10 mg/mL lysostaphin was added to the samples and allowed to incubate for 45 min at 37 °C. Finally, to detect pyruvate dehydrogenase complex protein, the samples were centrifuged at 10,000× *g* for 10 min at 4 °C. *Escherichia coli* was used as a positive control because pyruvate dehydrogenase complex activity has been shown for this species [59,60]. To assay in *E. coli*, the same process as above was done with the following changes: *E. coli* was grown on tryptic soy agar and 50 μL of 1 mg/mL lysozyme (Sigma-Aldrich) was used for cell lysis instead of lysostaphin [59,61].

For the assays, a 100 mL aliquot of each supernatant (*S. aureus* and *E. coli*) was added to a microtiter plate in triplicate with 200 μL of reaction mixture (2.5 mM NAD, 0.2 mM thiamine pyrophosphate, 0.1 mM coenzyme A, 0.3 mM dithiothreitol, 5 mM pyruvate, 1 mM magnesium chloride, and 0.05 M potassium phosphate buffer with pH adjusted to 6.6).

Reaction mixture with no pyruvate in triplicate was used as a blank. Negative control wells contained 200 μL of reaction mixture without sample. The samples were incubated at room temperature for five minutes before a SpectraMax M3 plate reader (Molecular Devices, San Jose, CA, USA) was used to take absorbance readings at 340 nm [26]. Enzyme activity was determined based on the molar extinction coefficient of NADH at 340 nm (6.22 mM^−1^ cm^−1^). The mean and standard deviation of three separate trials was calculated and a Student’s *t*-test was performed to measure statistical significance of averaged values with a *p*-value < 0.05 was considered statistically significant.

## Figures and Tables

**Table 1:** Genes mapping to KEGG metabolic pathways that displayed higher or lower transcript abundance differences in RNA-Seq comparing *S. aureus* Newman to *S. aureus* Newman *brpR*.

Gene	Log2-Fold Change	Gene Product
**Citrate cycle (TCA cycle**)		
*budA*	−1.08	Acetolactate decarboxylase
*fumC*	−1.04	Class II fumarate hydratase
*icd*	−1.00	NADP-dependent isocitrate dehydrogenase
*lpdA*	−1.31	Dihydrolipoyl dehydrogenase
*pdhA*	−1.20	Pyruvate dehydrogenase (acetyl-transferring) E1 component subunit alpha
*pdhC*	−1.29	Dihydrolipoamide acetyltransferase family protein
*sucB*	−1.13	Dihydrolipoyllysine-residue succinyltransferase
**Phosphotransferase system**		
*MW_RS01615*	1.30	PTS ascorbate transporter subunit IIC
*MW_RS01620*	1.45	PTS ascorbate transporter subunit IIB
*MW_RS01625*	1.45	PTS ascorbate transporter subunit IIA
*MW_RS03595*	−1.45	Fructose-specific PTS transporter subunit EIIC
**Quorum sensing**		
*MW_RS03175*	1.12	DMT family transporter
*MW_RS04875*	2.03	Competence protein ComK
*MW_RS06735*	2.06	Anthranilate synthase component
*MW_RS06740*	2.50	Aminodeoxychorismate/anthranilate synthase component II
*MW_RS09115*	−1.28	MarR family transcriptional regulator
*MW_RS10245*	−1.59	Phospholipase
*MW_RS10925*	1.20	RR transcription factor
**TCS**		
*lrgA*	1.52	Antiholin-like murein hydrolase modulator LrgA
*lrgB*	1.33	Antiholin-like protein LrgB
*MW_RS01030*	−1.31	RR transcription factor
*MW_RS01035*	−1.62	Sensor HK
*MW_RS01040*	−1.58	ABC transporter substrate-binding protein
*MW_RS05200*	1.10	Cytochrome ubiquinol oxidase subunit I
*MW_RS10925*	1.20	RR transcription factor
*MW_RS12555*	−2.13	Nitrate reductase subunit alpha
*narH*	−1.78	Nitrate reductase subunit beta
*narI*	−1.44	Respiratory nitrate reductase subunit gamma
*narJ*	−1.67	Nitrate reductase molybdenum cofactor assembly chaperone
*nreA*	−1.29	Nitrate respiration regulatory accessory nitrate sensor NreA
*nreB*	−1.37	Nitrate respiration regulation sensor HK NreB
*nreC*	−1.92	Nitrate respiration regulation RR NreC
**ABC transporters**		
*MW_RS03235*	−2.64	Metal ABC transporter substrate-binding protein
*MW_RS03240*	−3.32	Metal ABC transporter permease
*MW_RS03245*	−3.10	Metal ABC transporter ATP-binding protein
*MW_RS12745*	1.03	ABC transporter ATP-binding protein
*pstA*	1.22	Phosphate ABC transporter permease PstA
*pstB*	1.21	Phosphate ABC transporter ATP-binding protein PstB
*tagG*	−1.05	Teichoic acids export ABC transporter permease subunit TagG
*tnpA*	−1.19	IS200/IS605 family transposase
**Biosynthesis of nucleotide** **sugars**		
*galU*	−1.25	UTP-glucose-1-phosphate uridylyltransferase GalU
*MW_RS08020*	−1.31	ROK family glucokinase
**Carbon metabolism**		
*fumC*	−1.04	Class II fumarate hydratase
*icd*	−1.00	NADP-dependent isocitrate dehydrogenase
*lpdA*	−1.31	Dihydrolipoyl dehydrogenase
*MW_RS08020*	−1.32	ROK family glucokinase
*MW_RS12235*	−1.41	Ribose 5-phosphate isomerase A
*pdhA*	−1.20	Pyruvate dehydrogenase (acetyl-transferring) E1 component subunit alpha
*serA*	1.02	Phosphoglycerate dehydrogenase
*tdcB*	−3.75	Bifunctional threonine ammonia-lyase/L-serine ammonia-lyase TdcB
**Pyruvate metabolism**		
*fumC*	−1.04	Class II fumarate hydratase
*lpdA*	−1.31	Dihydrolipoyl dehydrogenase
*MW_RS05255*	−1.29	Dihydrolipoamide acetyltransferase family protein
*MW_RS13250*	−1.84	D-lactate dehydrogenase
*MW_RS13335*	1.35	Pyruvate oxidase
*pdhA*	−1.20	Pyruvate dehydrogenase (acetyl-transferring) E1 component subunit alpha
*plfB*	−1.57	Formate C-acetyltransferase
**D-amino acid metabolism**		
*alr*	1.72	Alanine racemase
**Peptidoglycan biosynthesis**		
*bacA*	−1.47	Undecaprenyl-diphosphate phosphatase
*mraY*	1.04	Phospho-N-acetylmuramoyl-pentapeptide- transferase
**Phenylalanine, tyrosine, and** **tryptophan biosynthesis**		
*MW_RS06735*	2.06	Anthranilate synthase component I
*MW_RS06755*	2.20	Phosphoribosylanthranilate isomerase
*MW_RS08985*	−1.32	Bifunctional 3-deoxy-7-phosphoheptulonate synthase/chorismate mutase
*trpA*	2.37	Tryptophan synthase subunit alpha
*trpB*	2.47	Tryptophan synthase subunit beta
*trpC*	3.06	Indole-3-glycerol phosphate synthase TrpC
*trpD*	2.86	Anthranilate phosphoribosyltransferase
**Cysteine and methionine** **metabolism**		
*metE*	−1.11	5-methyltetrahydropteroyltriglutamate- homocysteine S-methyltransferase
*MW_RS01755*	−1.21	Bifunctional homocysteine S- methyltransferase/methylenetetrahydrofolate reductase
*MW_RS01760*	−1.14	PLP-dependent aspartate aminotransferase family protein
*MW_RS01765*	−1.11	Aminotransferase class I/II-fold pyriodoxal phosphate-dependent enzyme
*serA*	1.02	Phosphoglycerate dehydrogenase
**Serine metabolism**		
*deoB*	−1.33	Phosphopentomutase
*MW_RS11980*	1.75	Urease subunit gamma
*ndk*	–1.25	Nucleoside-diphosphate kinase

**Table 2:** Primers used in this study for qRT-PCR analysis.

Primer Name	Sequence	Gene	Product Length	Reference
BacA3	5′ cctttaccaatgccaaatccg 3′	*bacA*	276 bp	This study
BacA4	5′ tcataaagcagcatcggacttt 3′			
CitC1	5′ ggaaagaagtgctagctggcc 3′	*icd*	234 bp	This study
CitC2	5′ gtggacgtttaacaggtgatggt 3′			
MetE1	5′ agatgtgcacgttcaaagtatg 3′	*metE*	361 bp	This study
MetE2	5′ gaatgggacaatgttgagccta 3′			
MraY1	5′ tcgagaagaaggtccacaaag 3′	*mraY*	352 bp	This study
MraY2	5′ tgatagtgggattgctacattc 3′			
MW1287-3	5′ aacaatcagccattaatggcag 3′	*alr*	240 bp	This study
MW1287-4	5′ gtaatatgtcaacgacggcaaa 3′			
PdhA1	5′ ggctcctaagttacaagc 3′	*pdhA*	179 bp	This study
PdhA2	5′ ggatacgagtccataccattc 3′			
SaFtsZ1	5′ ggtgtaggtggtggcggtaa 3′	*ftsZ*	484 bp	[51]
SaFtsZ2	5′ tcattggcgtagatttgtc 3′			

## Data Availability

The RNA-Seq data is available from the NCBI SRA database accession number PRJNA1495894. All other data is available upon request.

## Supplementary Materials

The following supporting information can be downloaded at: https://www.mdpi.com/article/10.3390/antibiotics15080787/s1, Table S1: Genes significantly upregulated or downregulated from RNA sequencing of wild-type *S. aureus* strain Newman compared to the Newman brpR mutant; Code used in R to download KEGG pathways.
