# Supplementary material for: Global Transcriptional Differences in Staphylococcus aureus Biofilm-Associated Genes in a brpR Mutant Compared to Wild-Type Strain
Source: Antibiotics (Basel). 2026 Aug 14;15(8):787. doi: 10.3390/antibiotics15080787 (PMC13509338; doi:10.3390/antibiotics15080787)
Supplement: Supplementary file 1 [file antibiotics-15-00787-s001.zip › antibiotics-4473468-supplementary.pdf]

Supplemental Table S1. Genes significantly upregulated or downregulated from RNA sequencing of wild type S. aureus strain Newman
compared to the Newman *brpR* mutant

Gene	Log_2-fold Change	*P*-value	Gene product	Pathways mapped to in KEGG
adhP	–1.09	0.00	Alcohol dehydrogenase AdhP	
ald	–1.27	0.00	Alanine dehydrogenase	Taurine and hypotaurine metabolism; alanine, aspartate and glutamate metabolism
alr	1.72	0.00	Alanine racemase	
als	–1.31	0.00	Acetolactate synthase AlsS	
argH	1.40	0.00	Argininosuccinate lyase	Alanine, aspartate and glutamate metabolism; arginine biosynthesis
bacA	–1.47	0.00	Undecaprenyl-diphosphate phosphatase	
bioA	1.24	0.00	Adenosylmethionine-8-amino-7-oxononanoate transaminase	Biosynthesis of cofactors; biotin metabolism
budA	4.73	0.00	Acetolactate decarboxylase	2-oxocarboxylic acid metabolism; C-5 branched dibasic acid metabolism; butanoate metabolism
clpB	–2.17	0.00	ATP-dependent chaperone ClpB	
clpP	–8.85	0.00	Clp protease ClpP	
cobA	–1.28	0.00	Uroporphyrinogen-III C-methyltransferase	
comGC	1.92	0.00	Competence type IV pilus major pilin ComGC	Biosynthesis of nucleotide sugars; teichoic acid biosynthesis; O-antigen nucleotide sugar biosynthesis

cstA	1.60	0.00	Persulfide response sulfurtransferase CstA	Biosynthesis of nucleotide sugars; O-antigen nucleotide sugar biosynthesis; amino sugar and nucleotide sugar metabolism
cstR	1.30	0.00	Persulfide-sensing transcriptional repressor CstR	Biosynthesis of nucleotide sugars; O-antigen nucleotide sugar biosynthesis
deoB	–1.33	0.00	Phosphopentomutase	Biosynthesis of nucleotide sugars; O-antigen nucleotide sugar biosynthesis
deoC	–1.04	0.00	Deoxyribose-phosphate aldolase	
deoD	–1.05	0.01	Purine-nucleoside phosphorylase	
dnaJ	–1.29	0.00	Molecular chaperone DnaJ	TCS; biosynthesis of nucleotide sugars; O-antigen nucleotide sugar biosynthesis; amino sugar and nucleotide sugar metabolism
dnaK	–1.47	0.00	Molecular chaperone DnaK	
efeB	1.46	0.00	Iron uptake transporter deferrochelatase/peroxidase	
ezrA	–1.30	0.00	Septation ring formation regulator EzrA	
farE	1.78	0.00	Fatty acid efflux MMPL transporter FarE	Biosynthesis of cofactors; Microbial metabolism in diverse environments; porphyrin metabolism
farR	2.65	0.00	Fatty acid efflux pump transcriptional regulator FarR	
fumC	–1.04	0.00	Class II fumarate hydratase	
galU	–1.26	0.00	UTP-glucose-1-phosphate uridylyltransferase GalU	
graF	1.51	0.00	Glycopeptide resistance-associated GraF	Purine metabolism; pentose phosphate pathway
groL	–1.18	0.00	Chaperonine GroEL	
grpE	–1.23	0.00	Nucleotide exchange factor GrpE	
harA	–5.39	0.00	Haptoglobin-binding heme uptake	

			protein HarA	
hemC	–1.11	0.00	Hydroxymethylbilane synthase	RNA degradation
hisA	1.09	0.00	1-(5-phosphoribosyl)-5-((5-phosphoribosylamino)methylideneamino)imidazole-4-carboxamide isomerase	
hisF	1.03	0.00	Imidazole glycerol phosphate synthase subunit HisF	
hrcA	–1.26	0.00	Heat-inducible transcriptional repressor HrcA	
hyl	–3.55	0.00	Alpha-hemolysin	
icd	–1.00	0.00	NADP-dependent isocitrate dehydrogenase	Citrate cycle (TCA cycle); carbon metabolism; microbial metabolism in diverse environments; pyruvate metabolism
isaB	–1.41	0.00	Immunodominant staphylococcal antigen IsaB	Biosynthesis of nucleotide sugars; biosynthesis of cofactors; O-antigen nucleotide sugar biosynthesis; amino sugar and nucleotide sugar metabolism; starch and sucrose metabolism; galactose metabolism; pentose and glucuronate interconversions
isdB	3.03	0.00	Heme uptake protein IsdB	
isdD	1.02	0.00	Iron-regulated surface determinant protein IsdD	RNA degradation
isdE	1.23	0.00	Heme ABC transporter substrate-binding protein IsdE	
isdF	1.35	0.00	Hemin ABC transporter permease protein IsdF	
lip2	–3.47	0.00	YSIRK domain-containing triacylglycerol lipase Lip2/Geh	Biosynthesis of cofactors; microbial metabolism in diverse environments; porphyrin metabolism
lpdA	–1.31	0.00	Dihydrolipoyl dehydrogenase	Histidine metabolism
lrgA	1.52	0.00	Antiholin-like murein hydrolase	

			modulator LrgA	
lrgB	1.33	0.01	Antiholin-like protein LrgB	
mdeA	1.56	0.00	Multidrug efflux MFS transporter MdeA	
menC	–1.13	0.00	o-succinylbenzoate synthase	
menE	–1.15	0.00	o-succinylbenzoate-CoA ligase	
mepR	2.22	0.00	Multidrug efflux transporter transcriptional repressor MepR	
metE	–1.11	0.00	5-methyltetrahydropteroyl triglutamate-homocysteine S-methyltransferase	
mnhA2	15.80	0.00	Na+/H+ antiporter Mnh2 subunit A	Glycerolipid metabolism
mnhB2	8.92	0.00	Na+/H+ antiporter Mnh2 subunit B	Glycolysis/Gluconeogenesis; TCA cycle; biosynthesis of cofactors; 2-oxocarboxylic acid metabolism; carbon metabolism; microbial metabolism in diverse environments; lipoic acid metabolism; propanoate metabolism; pyruvate metabolism; tryptophan metabolism; lysine degradation; valine, leucine and isoleucine degradation; glycine, serine and threonine metabolism
mnhC2	3.94	0.00	Na+/H+ antiporter Mnh2 subunit C	TCS
mnhD2	5.16	0.00	Na+/H+ antiporter Mnh2 subunit D	TCS
mnhE2	4.87	0.00	Na+/H+ antiporter Mnh2 subunit E	
mnhF2	3.88	0.00	Na+/H+ antiporter Mnh2 subunit F	
mnhG2	4.29	0.00	Na+/H+ antiporter Mnh2 subunit G	
mraY	1.04	0.00	Phospho-N-acetylmuramoyl-pentapeptide-transferase	Biosynthesis of cofactors; ubiquinone and other terpenoid-quinone biosynthesis
msaC	–1.21	0.00	*sarA* expression modulator MsaC	Biosynthesis of cofactors; ubiquinone and other terpenoid-quinone biosynthesis
mscL	–1.23	0.00	Large conductance mechano sensitive channel protein MscL	

MW_RS00135	–16.94	0.00	DUF3578 domain-containing protein	Selenocompound metabolism; cysteine and methionine metabolism
MW_RS00145	–14.55	0.00	IS6-like element IS257 family transposase	
MW_RS00155	–13.40	0.00	Glycerophosphoryl diester phosphodiesterase	
MW_RS00160	–8.41	0.00	MaoC family dehydratase	
MW_RS00170	–13.79	0.00	Beta-lactam sensor/signal transducer MecR1	
MW_RS00175	–13.25	0.00	DUF3387 domain-containing protein	
MW_RS00180	–2.94	0.00	IS1182 family transposase	
MW_RS00185	–11.16	0.00	DUF1643 domain-containing protein	
MW_RS00190	–9.62	0.00	DUF960 family protein	Peptidoglycan biosynthesis
MW_RS00195	–9.60	0.00	SAUGI family uracil-DNA glycosylase inhibitor	
MW_RS00200	–14.22	0.00	Recombinase family protein	
MW_RS00205	–9.69	0.00	Recombinase family protein	
MW_RS00210	–14.25	0.00	DUF927 domain-containing protein	
MW_RS00215	–11.17	0.00	Hypothetical protein	Glycerophospholipid metabolism
MW_RS00220	–15.43	0.00	Hypothetical protein	
MW_RS00225	–14.33	0.00	Putative DNA binding domain-containing protein	Beta-lactam resistance
MW_RS00240	–7.35	0.00	HNH endonuclease	
MW_RS00245	–16.87	0.00	Hypothetical protein	
MW_RS00250	–6.14	0.00	DUF3578 domain-containing protein	
MW_RS00410	1.36	0.00	M20 family metallopeptidase	
MW_RS00420	1.12	0.01	Na/Pi cotransporter family protein	
MW_RS00425	1.16	0.01	Oleate hydratase	

MW_RS00525	–1.28	0.00	(S)-acetoin forming diacetyl reductase	
MW_RS00565	–1.31	0.00	Hypothetical protein	
MW_RS00625	–1.08	0.00	Bifunctional UDP-sugar hydrolase/ 5'-nucleotidase	
MW_RS00645	–6.37	0.00	Hypothetical protein	
MW_RS00755	1.06	0.01	Cation diffusion facilitator family transporter	
MW_RS00770	–1.99	0.00	ABC transporter ATP-binding protein	
MW_RS00775	–1.95	0.00	ABC transporter substrate-binding protein	
MW_RS00780	–2.60	0.00	ABC transporter permease	
MW_RS00785	–2.23	0.00	Acyl-CoA/acyl-ACP dehydrogenase	
MW_RS00790	–1.61	0.00	DUF2294 domain-containing protein	
MW_RS00815	–1.20	0.00	YagU family protein	
MW_RS01005	–1.16	0.00	Gfo/Idh/MocA family oxidoreductase	Butanoate metabolism
MW_RS01010	–1.13	0.00	Gfo/Idh/MocA family oxidoreductase	
MW_RS01015	–1.32	0.00	Sugar phosphate isomerase/epimerase	
MW_RS01020	–3.63	0.00	Isoprenylcysteine carboxyl methyltransferase family protein	
MW_RS01030	–1.31	0.00	RR transcription factor	
MW_RS01035	–1.63	0.00	Sensor HK	
MW_RS01040	–1.58	0.00	ABC transporter substrate-binding protein	
MW_RS01065	–1.08	0.00	Complement inhibitor SCIN family protein	
MW_RS01135	1.03	0.00	Hypothetical protein	
MW_RS01265	–1.81	0.00	GntR family transcriptional regulator	
MW_RS01310	1.10	0.00	LacI family DNA-binding transcriptional regulator	
MW_RS01355	–3.93	0.00	Hypothetical protein	
MW_RS01360	–1.14	0.00	CHAP domain-containing protein	
MW_RS01420	1.33	0.00	TIGR01741 family protein	
MW_RS01465	1.92	0.00	TIGR01741 family protein	

MW_RS01475	1.04	0.00	DUF4467 domain-containing protein	TCS
MW_RS01560	1.55	0.00	YjiH family protein	TCS
MW_RS01570	1.04	0.00	Alpha/beta hydrolase	TCS
MW_RS01610	1.32	0.00	Aldehyde reductase	
MW_RS01615	1.30	0.00	PTS ascorbate transporter subunit IIC	
MW_RS01620	1.45	0.00	PTS sugar transporter subunit IIB	
MW_RS01625	1.45	0.00	PTS sugar transporter subunit IIA	
MW_RS01630	1.27	0.00	BglG family transcription antiterminator	
MW_RS01680	1.29	0.00	EfeM/EfeO family lipoprotein	
MW_RS01690	1.02	0.00	FTR1 family iron permease	
MW_RS01755	–1.21	0.00	Bifunctional homocysteine S-methyltransferase/methylene tetrahydrofolate reductase	
MW_RS01760	–1.14	0.00	PLP-dependent aspartate aminotransferase family protein	
MW_RS01765	–1.11	0.00	Aminotransferase class I/II-fold pyriodoxal phosphate-dependent enzyme	
MW_RS01770	1.13	0.00	ParB/RepB/Spo0J family partition protein	
MW_RS01810	1.58	0.00	Abi family protein	
MW_RS01860	–1.14	0.00	NDxxF motif lipoprotein	Phosphotransferase system (PTS); microbial metabolism in diverse environments; ascorbate and aldarate metabolism
MW_RS01895	–1.53	0.00	Hypothetical protein	PTS
MW_RS02010	1.20	0.00	Superantigen-like protein SSL1	PTS
MW_RS02030	1.19	0.00	Superantigen-like protein SSL4	
MW_RS02085	–3.12	0.00	Superantigen-like protein SSL11	
MW_RS02090	–2.18	0.00	FKLRK protein	
MW_RS02135	–1.34	0.00	Hypothetical protein	Microbial metabolism in diverse environments; one carbon pool by folate; cysteine and methionine metabolism
MW_RS02350	–1.17	0.00	DNA polymerase III subunit delta’	Selenocompound metabolism;

				cysteine and methionine metabolism
MW_RS02405	–1.14	0.00	Veg family protein	Sulfur metabolism; selenocompound metabolism; cysteine and methionine metabolism
MW_RS02640	–1.74	0.00	CtsR family transcriptional regulator	
MW_RS02645	–1.77	0.00	UvrB/UvrC motif-containing protein	
MW_RS02650	–1.82	0.00	Protein arginine kinase	
MW_RS02655	–1.72	0.00	ATP-dependent Clp protease ATP-binding subunit	
MW_RS02740	–1.12	0.00	Class I SAM-dependent methyltransferase	
MW_RS02860	–1.09	0.00	Glycosyltransferase	
MW_RS02900	1.11	0.00	MFS transporter	
MW_RS02905	1.17	0.00	Long-chain fatty acid-CoA ligase	
MW_RS02910	1.16	0.00	Thiolase family protein	
MW_RS02920	1.48	0.00	Hypothetical protein	DNA replication
MW_RS03115	–2.45	0.00	Hypothetical protein	
MW_RS03175	1.12	0.00	DMT family transporter	
MW_RS03180	8.21	0.00	DUF2922 domain-containing protein	
MW_RS03185	8.26	0.00	DUF1659 domain-containing protein	
MW_RS03190	12.13	0.00	Tyrosine-type recombinase/integrase	
MW_RS03235	–2.79	0.00	Metal ABC transporter substrate-binding protein	
MW_RS03240	–3.32	0.00	Metal ABC transporter permease	
MW_RS03245	–3.10	0.00	Metal ABC transporter ATP-binding protein	
MW_RS03275	–1.49	0.00	CDP-glycerol glycerophosphotransferase family protein	
MW_RS03280	–1.50	0.00	Glycosyltransferase family A protein	
MW_RS03475	1.01	0.00	GNAT family N-acetyltransferase	
MW_RS03540	–1.24	0.00	Hypothetical protein	
MW_RS03545	–1.30	0.00	DASS family sodium-coupled anion symporter	
MW_RS03595	–1.45	0.00	Fructose-specific PTS transporter subunit EIIC	

MW_RS03665	3.21	0.00	Aminodeoxychorismate/anthranilate synthase component II	
MW_RS03670	3.34	0.00	Anthranilate synthase component I family protein	ABC transporter
MW_RS03675	3.34	0.00	Aminotransferase class IV	ABC transporter
MW_RS03680	2.14	0.00	Hypothetical protein	ABC transporter
MW_RS03685	2.11	0.00	Allophanate hydrolase subunit I	Teichoic acid biosynthesis
MW_RS03690	1.87	0.00	Biotin-dependent carboxyltransferase family protein	
MW_RS03970	1.24	0.00	YvcK family protein	
MW_RS03995	1.06	0.00	DUF4887 domain-containing protein	Teichoic acid biosynthesis; peptidoglycan biosynthesis
MW_RS04080	–12.76	0.00	DUF1474 family protein	
MW_RS04085	–15.59	0.00	Phage/plasmid primase, P4 family	
MW_RS04090	–13.62	0.00	Hypothetical protein	PTS; microbial metabolism in diverse environments; fructose and mannose metabolism
MW_RS04095	–12.05	0.00	Hypothetical protein	Biosynthesis of cofactors; folate biosynthesis
MW_RS04100	–16.12	0.00	Phage capsid protein	
MW_RS04105	–12.11	0.00	Hypothetical protein	Biosynthesis of cofactors; folate biosynthesis
MW_RS04120	–12.49	0.00	DUF4888 domain-containing protein	
MW_RS04145	1.50	0.00	Hypothetical protein	
MW_RS04175	–1.83	0.00	Thermonuclease family protein	
MW_RS04225	1.83	0.00	LysE/ArgO family amino acid transporter	
MW_RS04250	1.09	0.00	Thioredoxin family protein	
MW_RS04275	1.18	0.00	Toprim domain-containing protein	
MW_RS04280	1.16	0.00	Thioredoxin family protein	
MW_RS04305	1.31	0.00	DUF368 domain-containing protein	
MW_RS04355	–1.30	0.00	DUF72 domain-containing protein	
MW_RS04365	–1.11	0.00	Bifunctional UDP-sugar hydrolase/ 5'-nucleotidase	

MW_RS04385	–1.75	0.00	DUF86 domain-containing protein	
MW_RS04395	–1.22	0.00	D-glycerate dehydrogenase	
MW_RS04555	1.27	0.00	Argininosuccinate synthase	
MW_RS04655	–1.17	0.00	YjzD family protein	
MW_RS04760	–1.20	0.00	CYTH domain-containing protein	
MW_RS04815	–1.34	0.00	YjcG family protein	
MW_RS04875	2.03	0.00	Competence protein ComK	
MW_RS04880	–1.70	0.00	IDEAL domain-containing protein	
MW_RS04935	–1.09	0.00	Hypothetical protein	
MW_RS04940	–1.07	0.00	Glycosyltransferase	
MW_RS05035	1.12	0.00	GNAT family N-acetyltransferase	
MW_RS05200	1.10	0.00	Cytochrome ubiquinol oxidase subunit I	Microbial metabolism in diverse environments; glyoxylate and dicarboxylate metabolism
MW_RS05210	1.19	0.00	TrkA family potassium uptake protein	Alanine, aspartate and glutamate metabolism; arginine biosynthesis
MW_RS05250	–1.20	0.00	Alpha-ketoacid dehydrogenase subunit beta	
MW_RS05305	4.11	0.00	Nramp family divalent metal transporter	
MW_RS05470	2.13	0.00	Hypothetical protein	
MW_RS05650	1.33	0.00	Superantigen-like protein SSL14	Quorum sensing
MW_RS06380	1.24	0.00	MerR family transcriptional regulator	
MW_RS06475	1.18	0.00	ABC transporter ATP-binding protein	
MW_RS06500	1.12	0.00	Thermonuclease family protein	Teichoic acid biosynthesis
MW_RS06595	1.38	0.00	DUF896 domain-containing protein	
MW_RS06680	1.03	0.00	Alanine/glycine:cation symporter family protein	TCS; Oxidative phosphorylation
MW_RS06710	1.19	0.00	LCP family protein	
MW_RS06735	2.06	0.00	Anthranilate synthase component I	
MW_RS06740	2.50	0.00	Aminodeoxychorismate/anthranilate	Glycolysis/Gluconeogenesis;

			synthase component II	TCA cycle; lipoic acid metabolism; pyruvate metabolism
MW_RS06755	2.20	0.00	Phosphoribosylanthranilate isomerase	
MW_RS06900	1.14	0.00	Amidohydrolase	
MW_RS06975	–1.43	0.00	DUF6501 family protein	
MW_RS06980	–1.37	0.00	Hypothetical protein	
MW_RS07005	2.13	0.00	Hypothetical protein	
MW_RS07125	–4.05	0.00	Amino acid permease	
MW_RS07150	2.20	0.00	PepSY domain-containing protein	
MW_RS07390	1.83	0.00	DUF1672 domain-containing protein	
MW_RS07395	1.42	0.00	DUF1672 domain-containing protein	Quorum sensing; phenylalanine, tyrosine and tryptophan biosynthesis
MW_RS07400	1.10	0.00	DUF1672 domain-containing protein	Quorum sensing
MW_RS07405	4.63	0.00	Hypothetical protein	Phenylalanine, tyrosine and tryptophan biosynthesis
MW_RS07420	6.31	0.00	N-acetylmuramoyl-L-alanine amidase	
MW_RS07425	8.44	0.00	Phage holin	D-amino acid metabolism
MW_RS07430	` 5.09	0.00	DUF2951 domain-containing protein	
MW_RS07440	–7.76	0.00	DUF2977 domain-containing protein	
MW_RS07445	–10.06	0.00	BppU family phage baseplate upper protein	
MW_RS07450	–11.48	0.00	Minor structural protein	
MW_RS07455	–8.04	0.00	Hypothetical protein	
MW_RS07460	–11.70	0.00	Prophage endopeptidase tail family protein	
MW_RS07465	–10.39	0.00	Phage tail family protein	
MW_RS07470	–14.07	0.00	Phage tail tape measure protein	
MW_RS07480	–5.14	0.00	Hypothetical protein	
MW_RS07485	–10.96	0.00	Ig-like domain-containing protein	
MW_RS07490	–9.69	0.00	Tail protein	
MW_RS07495	–7.86	0.00	DUF3168 domain-containing protein	
MW_RS07500	–9.03	0.00	Hypothetical protein	
MW_RS07505	–7.60	0.00	Hypothetical protein	

MW_RS07510	–8.18	0.00	Head-tail connector protein
MW_RS07515	–10.91	0.00	Phage major capsid protein
MW_RS07525	–11.10	0.00	Phage portal protein
MW_RS07530	–9.37	0.00	Terminase large subunit
MW_RS07535	–5.65	0.00	P27 family phage terminase small subunit
MW_RS07545	–12.46	0.00	Transcriptional regulator
MW_RS07550	–13.96	0.00	DEAD/DEAH box helicase
MW_RS07555	–10.20	0.00	VRR-NUC domain-containing protein
MW_RS07560	–9.39	0.00	Hypothetical protein
MW_RS07565	–13.94	0.00	VapE family protein
MW_RS07570	–10.44	0.00	DUF1514 family protein
MW_RS07580	12.66	0.00	Hypothetical protein
MW_RS07585	1.82	0.00	DUF1381 domain-containing protein
MW_RS07590	–12.79	0.00	Hypothetical protein
MW_RS07595	6.29	0.00	dUTPase
MW_RS07605	7.37	0.00	DUF1024 family protein
MW_RS07615	–7.29	0.00	Hypothetical protein
MW_RS07620	6.16	0.00	DUF3310 domain-containing protein
MW_RS07625	7.46	0.00	PVL family protein
MW_RS07635	–9.92	0.00	DNA polymerase
MW_RS07640	–8.20	0.00	DUF2815 domain-containing protein
MW_RS07645	–8.40	0.00	DUF2800 domain-containing protein
MW_RS07650	–5.75	0.00	Hypothetical protein
MW_RS07660	10.62	0.00	DUF1270 family protein
MW_RS07665	7.42	0.00	Helix-turn-helix domain-containing protein
MW_RS07670	–7.03	0.00	Hypothetical protein
MW_RS07695	1.55	0.00	Hypothetical protein
MW_RS07700	–7.84	0.00	Hypothetical protein
MW_RS07720	1.88	0.00	Hypothetical protein
MW_RS07760	1.07	0.01	DUF309 domain-containing protein
MW_RS07820	–1.12	0.00	LacI family DNA-binding transcriptional regulator
MW_RS07860	–1.04	0.00	Dihydrolipoamide acetyltransferase

			family protein	
MW_RS08010	–1.25	0.00	MBL fold metallo-hydrolase	
MW_RS08015	–1.05	0.00	MTH1187 family thiamine-binding protein	
MW_RS08020	–1.32	0.00	ROK family glucokinase	
MW_RS08025	–1.53	0.00	YqgQ family protein	
MW_RS08030	–1.42	0.00	Rhomboid family intramembrane serine protease	
MW_RS08035	–1.27	0.00	5-formyltetrahydrofolate cyclo-ligase	
MW_RS08055	1.04	0.00	Fur family transcriptional regulator	
MW_RS08680	–1.17	0.00	Hypothetical protein	
MW_RS08800	–1.02	0.00	Universal stress protein	
MW_RS08825	–1.76	0.00	Universal stress protein	
MW_RS08895	1.16	0.00	Alanine-glyoxylate aminotransferase family protein	Lipoic acid metabolism; propanoate metabolism; valine, leucine and isoleucine biosynthesis
MW_RS08985	–1.32	0.00	Bifunctional 3-deoxy-7-phosphoheptulonate synthase/chorismate mutase	
MW_RS09025	–1.13	0.00	Thioredoxin family protein	
MW_RS09030	–1.07	0.00	M42 family metallopeptidase	Glycolysis/Gluconeogenesis; biosynthesis of nucleotide sugars; carbon metabolism; microbial metabolism in diverse environments; streptomycin biosynthesis; amino sugar and nucleotide sugar metabolism; starch and sucrose metabolism; galactose metabolism
MW_RS09115	–1.28	0.00	MarR family transcriptional regulator	
MW_RS09160	1.39	0.00	Metalloregulator ArsR/SmtB family transcription factor	
MW_RS09240	–1.12	0.00	Prolyl oligopeptidase family serine peptidase	One carbon pool by folate
MW_RS09445	–1.21	0.00	DUF1828 domain-containing protein	

MW_RS09825	–1.20	0.00	Aromatic acid exporter family protein	
MW_RS09850	–1.90	0.00	Metal-dependent hydrolase	
MW_RS10245	–1.59	0.00	Phospholipase	
MW_RS10270	1.60	0.00	SH3 domain-containing protein	
MW_RS10285	–2.72	0.00	CHAP domain-containing protein	Phenylalanine, tyrosine, and tryptophan biosynthesis
MW_RS10290	–3.38	0.00	Phage holin	
MW_RS10310	11.27	0.00	Hypothetical protein	
MW_RS10315	9.54	0.00	Hypothetical protein	Quorum sensing
MW_RS10420	–1.80	0.00	DUF1514 family protein	
MW_RS10440	2.29	0.00	Transcriptional activator RinB	
MW_RS10450	–3.94	0.00	SA1788 family PVL leucocidin-associated protein	
MW_RS10455	2.79	0.00	RusA family crossover junction endodeoxyribonuclease	
MW_RS10460	–1.92	0.00	Hypothetical protein	
MW_RS10465	–3.50	0.00	DnaD domain-containing protein	Quorum sensing; glycerophospholipid metabolism; inositol phosphate metabolism
MW_RS10475	–2.56	0.00	MBL fold metallo-hydrolase	
MW_RS10480	–2.39	0.00	Recombinase RecT	
MW_RS10485	–2.41	0.00	AAA family ATPase	
MW_RS10490	–3.06	0.00	Hypothetical protein	
MW_RS10500	12.14	0.00	DUF1270 domain-containing protein	
MW_RS10510	12.88	0.00	Hypothetical protein	
MW_RS10515	8.78	0.00	Hypothetical protein	
MW_RS10520	9.64	0.00	Hypothetical protein	
MW_RS10560	–1.27	0.00	Site-specific integrase	
MW_RS10600	1.55	0.00	GNAT family N-acetyltransferase	
MW_RS10605	1.72	0.00	Hypothetical protein	
MW_RS10610	–1.02	0.00	Terminase small subunit	
MW_RS10700	1.09	0.00	Ammonium transporter	
MW_RS10855	1.31	0.00	PP2C family protein-serine/threonine	

			phosphatase	
MW_RS10925	1.20	0.00	RR transcription factor	
MW_RS11215	–1.00	0.00	Pyrimidine-nucleoside phosphorylase	
MW_RS11390	1.60	0.00	Hypothetical protein	
MW_RS11415	–1.36	0.00	Hemolysin III family protein	
MW_RS11495	1.42	0.00	BCCT family transporter	
MW_RS11555	–1.01	0.00	NAD-dependent protein deacylase	
MW_RS11585	1.27	0.00	Hypothetical protein	
MW_RS11590	1.45	0.00	Polysaccharide lyase 8 family protein	
MW_RS11595	1.49	0.00	MAP domain-containing protein	
MW_RS11610	1.04	0.00	Hypothetical protein	
MW_RS11810	–1.30	0.00	GNAT family N-acetyltransferase	
MW_RS11970	1.45	0.00	Acyl-CoA dehydrogenase family protein	Quorum sensing; TCS
MW_RS11980	1.75	0.00	Urease subunit gamma	Nucleotide metabolism; pyrimidine metabolism
MW_RS11985	1.42	0.00	Urease subunit beta	
MW_RS12065	1.73	0.00	CHAP domain-containing protein	
MW_RS12075	–1.15	0.00	2-hydroxyacid dehydrogenase family protein	
MW_RS12085	–1.69	0.00	N-acetylglucosaminidase	
MW_RS12235	–1.41	0.00	Ribose 5-phosphate isomerase A	
MW_RS12355	1.49	0.00	YdcF family protein	
MW_RS12380	1.05	0.00	LytTR family DNA-binding domain-containing protein	
MW_RS12385	1.19	0.00	DUF3021 domain-containing protein	
MW_RS12405	1.17	0.00	Hypothetical protein	
MW_RS12450	1.04	0.00	YhgE/Pip domain-containing protein	
MW_RS12470	1.23	0.00	YbfA family protein	Microbial metabolism in diverse environments; purine metabolism; arginine biosynthesis
MW_RS12555	–2.13	0.00	Nitrate reductase subunit alpha	
MW_RS12575	–1.20	0.00	Sirohydrochlorin chelatase	
MW_RS12590	–2.01	0.00	Hypothetical protein	

MW_RS12605	–1.74	0.00	Txe/YoeB family addiction module toxin	
MW_RS12610	–1.79	0.00	Type II toxin-antitoxin system Phd/YefM family antitoxin	Carbon metabolism; microbial metabolism in diverse environments; pentose phosphate pathway
MW_RS12665	–1.02	0.00	2,3-diphosphoglycerate-dependent phosphoglycerate mutase	
MW_RS12675	–1.83	0.00	Hypothetical protein	
MW_RS12745	1.03	0.00	ABC transporter ATP-binding protein	
MW_RS12750	1.16	0.00	ABC transporter ATP-binding protein	
MW_RS12935	–1.19	0.00	FMN-binding glutamate synthase family protein	
MW_RS12945	–1.70	0.00	MFS transporter	
MW_RS12990	1.28	0.00	Hypothetical protein	TCS
MW_RS12995	1.32	0.00	SDR family oxidoreductase	
MW_RS13020	–1.13	0.00	DUF1422 domain-containing protein	
MW_RS13050	–1.27	0.00	Hypothetical protein	
MW_RS13070	1.18	0.00	Tandem-type lipoprotein	
MW_RS13110	–1.97	0.00	E domain-containing protein	
MW_RS13165	–1.35	0.00	MerR family transcriptional regulator	
MW_RS13170	–1.28	0.00	GTP pyrophosphokinase family protein	ABC transporter
MW_RS13180	1.11	0.00	FUSC family protein	
MW_RS13245	–1.44	0.00	NAD(P)H-dependent oxidoreductase	
MW_RS13250	–1.84	0.00	D-lactate dehydrogenase	
MW_RS13285	1.35	0.00	Hypothetical protein	
MW_RS13300	1.48	0.00	PTS fructose transporter subunit IIC	
MW_RS13335	1.35	0.00	Pyruvate oxidase	
MW_RS13340	1.73	0.00	LrgB family protein	
MW_RS13360	1.21	0.00	CHAP domain-containing protein	
MW_RS13440	–1.02	0.00	D-lactate dehydrogenase	
MW_RS13495	3.60	0.00	PTS transporter subunit IIC	
MW_RS13550	1.08	0.00	Amidohydrolase family protein	
MW_RS13795	1.36	0.00	Hypothetical protein	

MW_RS13885	1.99	0.00	Arginine repressor	
MW_RS14140	1.56	0.00	YceI family protein	Microbial metabolism in diverse environments; pyruvate metabolism
MW_RS14230	–1.29	0.00	DUF3147 family protein	
MW_RS14335	–11.52	0.00	Nucleoid-structuring protein H-NS	
MW_RS14580	–11.84	0.00	Terminase small subunit	
MW_RS14860	–1.09	0.00	Site-specific integrase	
MW_RS15005	3.74	0.00	Hypothetical protein	
MW_RS15220	–13.51	0.00	Hypothetical protein	
MW_RS15275	–1.35	0.00	LPXTG cell wall anchor domain-containing protein	
MW_RS15395	–1.52	0.00	E domain-containing protein	
narH	–1.78	0.00	Nitrate reductase subunit beta	
narI	–1.44	0.00	Respiratory nitrate reductase subunit gamma	
narJ	–1.67	0.00	Nitrate reductase molybdenum cofactor assembly chaperone	
ndk	–1.25	0.00	Nucleoside-diphosphate kinase	
nirB	–1.34	0.00	Nitrate reductase large subunit NirB	
nirD	–1.34	0.00	Nitrate reductase small subunit NirD	
norB	–3.21 `	0.00	Multidrug efflux MFS transporter NorB	
norC	1.30	0.00	Multidrug efflux MFS transporter NorC	
nreA	–1.29	0.00	Nitrate respiration regulatory accessory nitrate sensor NreA	
nreB	–1.37	0.00	Nitrate respiration regulation sensor HK NreB	
nreC	–1.92	0.00	Nitrate respiration regulation RR NreC	
pdhA	–1.20	0.00	Pyruvate dehydrogenase (acetyl-transferring) E1 component subunit alpha	TCS; nitrogen metabolism
pdhC	–1.29	0.00	Dihydrolipoamide acetyltransferase family protein	
pepA1	–1.75	0.00	Type I toxin-antitoxin system Fst family toxin PepA1	TCS; microbial metabolism in diverse environments; nitrogen

				metabolism
perR	–1.43	0.00	Peroxide-responsive transcriptional repressor PerR	TCS
pfkB	–1.61	0.00	1-phosphofructokinase	Biosynthesis of cofactors; nucleotide metabolism; pyrimidine metabolism; purine metabolism
pflA	–1.52	0.00	Pyruvate formate-lyase-activating protein	
pflB	–1.57	0.00	Formate C-acetyltransferase	Microbial metabolism in diverse environments; nitrogen metabolism
pmtB	–1.09	0.00	Phenol-soluble modulin export ABC transporter permease subunit PmtB	
pmtC	–1.05	0.00	Phenol-soluble modulin export ABC transporter ATP-binding protein PmtC	
pmtD	–1.05	0.00	Phenol-soluble modulin export ABC transporter permease subunit PmtD	TCS
polX	1.08	0.00	DNA polymerase/3’-5’ exonuclease PolX	TCS
prmA	–1.23	0.00	50S ribosomal protein L11 methyltransferase	TCS
pstA	1.22	0.00	Phosphate ABC transporter permease PstA	Glycolysis/Gluconeogenesis; TCA cycle; 2-oxocarboxylic acid metabolism; carbon metabolism; lipoic acid metabolism; pyruvate metabolism
pstB	1.21	0.00	Phosphate ABC transporter ATP-binding protein PstB	
raiA	–1.48	0.00	Ribosome-associated translation inhibitor RaiA	
rbsK	–1.03	0.00	Ribokinase	PTS; fructose and mannose metabolism
recN	1.12	0.00	DNA repair protein RecN	

rpsA	–1.68	0.00	30S ribosomal protein S1	Microbial metabolism in diverse environments; buanoate metabolism; propanoate metabolism; pyruvate metabolism
sarA	1.64	0.00	Global transcriptional regulator SarA	
sarS	–1.69	0.00	HTH-type transcriptional regulator SarS	
sarT	–2.15	0.00	HTH-type transcriptional regulator SarT	
sarX	2.10	0.00	HTH-type transcriptional regulator SarX	
sbi	–2.62	0.00	Immunoglobulin-binding protein Sbi	
sceD	2.77	0.00	Lytic transglycosylase SceD	ABC transporter
scn	–1.64	0.00	Complement inhibitor SCIN-A	ABC transporter
sdaAB	1.42	0.00	L-serine ammonia-lyase, iron-sulfur-dependent subunit beta	
sdrE	1.68	0.00	MSCRAMM family adhesin SdrE	Pentose phosphate pathway
sea	15.09	0.00	Staphylococcal enterotoxin type A	
secA2	1.13	0.00	Accessory Sec system translocase SecA2	Ribosome
sek	–12.69	0.00	Staphylococcal enterotoxin type K	
seq	–12.39	0.00	Staphylococcal enterotoxin type Q	
serA	1.02	0.00	Phosphoglycerate dehydrogenase	
serS	–1.67	0.00	Serine-tRNA ligase	
spa	–1.44	0.00	Staphylococcal protein A	
sph	–1.18	0.00	Sphingomyelin phosphodiesterase	
sucB	–1.13	0.00	Dihydrolipoyllysine-residue succinyltransferase	
tag	–1.05	0.00	Teichoic acids export ABC transporter permease subunit TagG	
tdcB	–3.75	0.00	Bifunctional threonine ammonia-lyase/ L-serine ammonia-lyase TdcB	
tet(38)	–1.68	0.00	Tetracycline efflux MFS transporter Tet(38)	
tnpA	–1.19	0.00	IS200/IS605 family transposase	
topA	–1.17	0.00	Type I DNA topoisomerase	
treC	–1.17	0.00	Alpha,alpha-phosphotrehalase	

trpA	2.37	0.00	Tryptophan synthase subunit alpha	Carbon metabolism; microbial metabolism in diverse environments; methane metabolism; cysteine and methionine metabolism; glycine, serine and threonine metabolism
trpB	2.47	0.00	Tryptophan synthase subunit beta	Aminoacyl-tRNA biosynthesis
trpC	3.06	0.00	Indole-3-glycerol phosphate synthase TrpC	
trpD	2.86	0.00	Anthranilate phosphoribosyltransferase	
ureC	1.29	0.00	Urease subunit alpha	TCA cycle; lipoic acid metabolism; lysine degradation
uvrB	–1.16	0.00	Excinuclease ABC subunit UvrB	ABC transporter
whiA	1.19	0.00	DNA-binding protein WhiA	Carbon metabolism; valine, leucine, and isoleucine biosynthesis; glycine, serine, and threonine metabolism
ybaK	–1.05	0.00	Cys-tRNA (Pro) deacylase	PTS; TCS; ABC transporter
yfkAB	1.61	0.00	Radical SAM/CxCxxxxC motif protein YfkAB	
ygs	–1.26	0.00	S1 domain-containing post-translational regulator Ygs	
yjbH	–1.37	0.00	Protease adaptor protein YjbH	Starch and sucrose metabolism; phenylalanine, tyrosine, and tryptophan biosynthesis; glycine, serine, and threonine metabolism;
yjbI	–1.07	0.00	Truncated hemoglobin YjbI	
